# The updated understanding of advanced maternal age

**DOI:** 10.1016/j.fmre.2023.09.013

**Published:** 2023-12-29

**Authors:** Xuan Ye, Philip N. Baker, Chao Tong

**Affiliations:** a National Clinical Research Center for Child Health and Disorder, Children's Hospital of Chongqing Medical University, Chongqing 401122, China; b State Key Laboratory of Maternal and Fetal Medicine of Chongqing Municipality, The First Affiliated Hospital of Chongqing Medical University, Chongqing 400016, China; c College of Life Sciences, University of Leicester, Leicester LE1 7RH, UK

**Keywords:** Aging, Advanced maternal age, Reproductive system, Pregnancy outcomes, AMA pregnancies

## Abstract

The rising rates of pregnancies associated with advanced maternal age (AMA) have created unique challenges for healthcare systems worldwide. The elevated risk of poor maternal outcomes among AMA pregnancies is only partially understood and hotly debated. Specifically, AMA is associated with reduced fertility and an increased incidence of pregnancy complications. Finding a balance between global fertility policy, socioeconomic development and health care optimization ultimately depends on female fertility. Therefore, there is an urgent need to develop technologies and identify effective interventions. Support strategies should include prepregnancy screening, intervention and postpartum maintenance. Although some reviews have considered the relationship between AMA and adverse pregnancy outcomes, no previous work has comprehensively considered the long-term health effects of AMA on mothers. In this review, we will begin by presenting the current knowledge of global health issues associated with AMA and the effects of advanced age on the female reproductive system, endocrine metabolism, and placental function. We will then discuss physiological alterations, pregnancy complications, and long-term health problems caused by AMA.

## 1 Introduction

The average age of motherhood has gradually increased globally in recent decades [1]; this has been due to various socioeconomic factors, including delayed marriage, the pursuit of advanced education and careers, and higher rates of divorce and remarriage, as well as the development of assisted reproductive technologies [2,3]. Women conceived at older age are more likely to suffer from a range of pregnancy complications, including hypertensive disorders during pregnancy, gestational diabetes, postpartum hemorrhage, caesarean sections, iatrogenic and spontaneous preterm births [2]. In general, AMA is defined as a female age of 35 years or more at the time of a full-term birth [4,5]. However, the AMA-defining age of 35 years has been increasing to 40 or 45 years, and even older—often with the added description of ‘‘very advanced’’ [6,7], to better differentiate between those who have more recently crossed the AMA threshold and those who are pregnant much later in life.

Delaying childbirth can have relevant and beneficial effects on children's education and women's economic and emotional ability, better preparing individuals to raise children [4]; however, there are also clinical and public health risks [7]. Based on centuries-old observations, advanced age is an independent influencing factor on adverse pregnancy outcomes [8], such as premature delivery, cesarean section, preeclampsia, postpartum hemorrhage, and gestational diabetes mellitus [9]. These studies clearly show that AMA-associated pregnancies pose a particular global health challenge and that advanced-age women are a very important but under-researched group of pregnant women. In this review, we summarize the current understanding of AMA, and consider relevant and urgent problems related to AMA that need further exploration.

## 2 Impact of AMA on the female reproductive system

### 2.1 Uterus

#### 2.1.1 Implantation and decidualization

The implantation of the embryo in the uterus is the first step of a successful pregnancy. The coordinated action of progesterone and estrogen secreted by the corpus luteum establishes the receptivity of the uterus to implantation. During the implantation process, uterine epithelial cells begin to undergo apoptosis [10].

It has been extensively shown that aging has incontrovertible and detrimental effects on the uterus, as the endometrium loses its ability to support implantation and growth of an embryo. Mice aged 12 months have been found to have a 15% reduction in the number of implantation sites in early pregnancy and a 50% reduction in fertility at term [11]. Surprisingly, placental and embryonic abnormalities can be rescued when fertilized embryos from older mice are transplanted into the uteri of younger mice to develop further [12]; conversely, reverse transfer of embryos from young donors into older recipients results in developmental delay [13]. In the aged uterus, abnormal methylation and expression of imprinted genes may reduce the successes of implantation [14].

Meanwhile, the stromal cells surrounding the implanted blastocyst proliferate rapidly and then differentiate into morphologically and functionally distinct cells [15] in the process of decidualization. It begins on the anti-uterine surface, forming a structure (decidua) that wraps around the fetus and placenta until the placenta is formed. Although the decidua is a transient tissue, one of its key functions is to enable implantation of the embryo and nourish it to ensure a successful pregnancy.

Estrogen stimulates the proliferation of uterine epithelial and stromal cells, whereas progesterone inhibits estrogen-mediated proliferation of luminal and glandular epithelial cells [10,15]. Progesterone from the newly formed corpus luteum then initiates stromal cell proliferation, which is further enhanced by preimplantation ovarian estrogen secretion on the implantation day [10]. In older female mice, the expression of estrogen and progesterone receptors in the endometrium decreases with age [16,17], as does progesterone secretion [15]; consequently, the uterus of the aged female also becomes less responsive to steroid hormones [16]. These changes affect the decidual response to a great extent, which inevitably leads to reproductive failure [18].

#### 2.1.2 Myometrial contractions

In vitro spontaneous contractions of myometrium isolated from pregnant and nonpregnant women decrease with maternal age [19]. Uterine contractility is a coordinated process, and the transition from the static myometrium to the active rhythmic contraction state requires complex interactions between the placenta, fetus and mother. As maternal age increases, the response efficiency of the muscle tissue to intrauterine enhancers (such as oxytocin or prostaglandin) decreases [20,21]. In addition, myometrial tissue responds less effectively to oxytocin in the nonpregnant state as maternal age increases [20,21]. Older mothers therefore have a higher demand for oxytocin [22]. After exclusion of other maternal characteristics (number of previous cesarean deliveries, height, and body mass index), age is the only maternal characteristic associated with polyphasic contractions. This means that increasing maternal age is associated with decreased spontaneous activity and an increased likelihood of polyphasic spontaneous myometrial contractions [19]. Consistently, increasing maternal age in C57BL/6 J mice not only prolongs gestational length but also delays progesterone withdrawal and compromises myometrial function [23]. However, a reliable clinical treatment for pregnant women with a reduced myometrial response to intrauterine enhancers due to advanced age has yet to be elucidated.

#### 2.1.3 Cervical tissues

The maturity of the cervix determines the success of induction and is therefore closely related to birth outcomes and reduced maternal trauma. Maternal age also affects cervical ripening. A retrospective cohort study showed that maternal age over 30 years was an independent predictor of prostaglandin (PG) E2 cervical maturity failure [24]. Cervical tissues from older mice are more distensible than those from younger mice [23].

### 2.2 Ovary

**Fig. 1:**
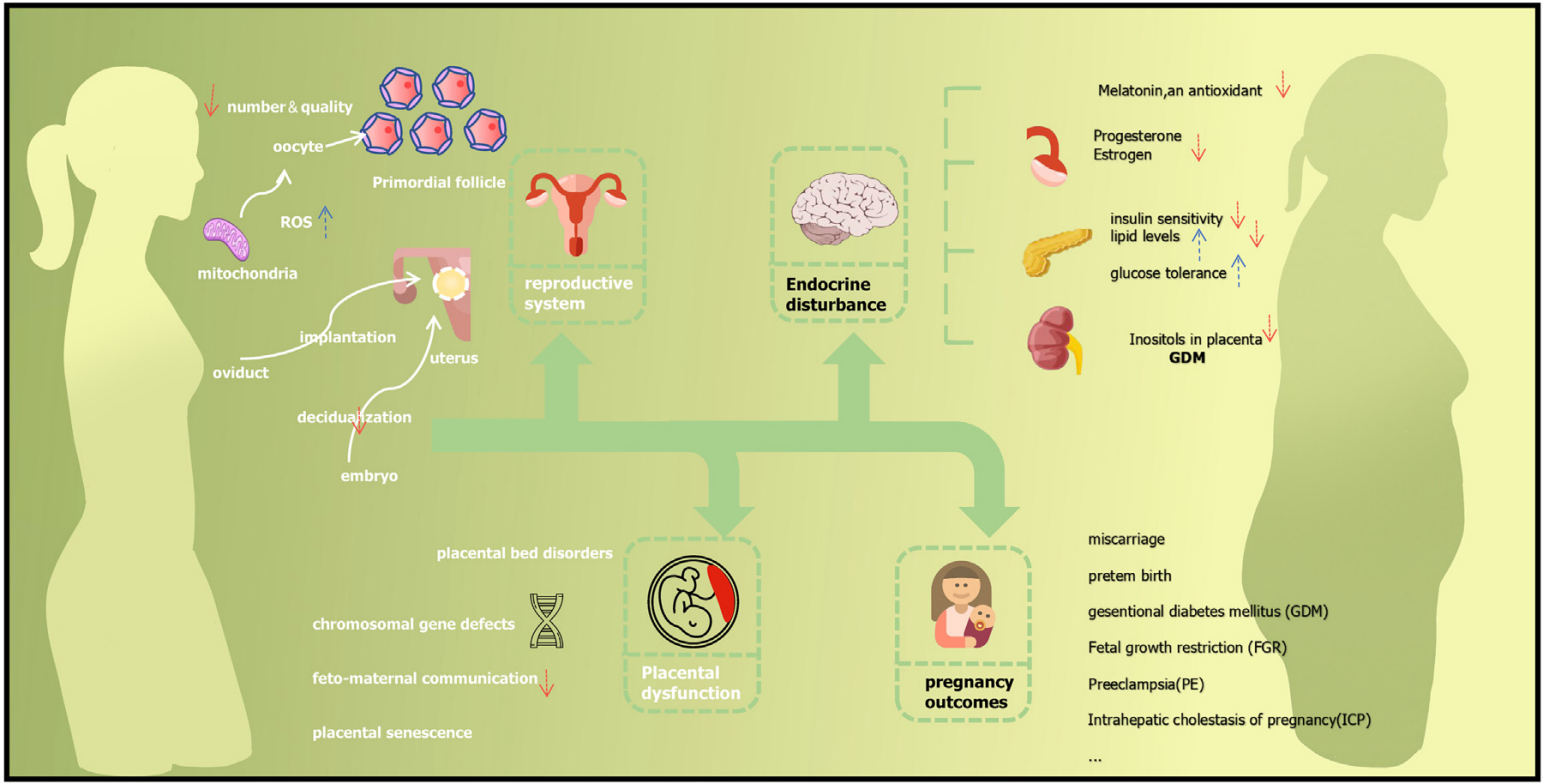
**The maternal and fetal outcomes of AMA**.

*Follicles and oocytes:* To date, the decline in ovarian function with age has been characterized by a gradual depletion of ovarian follicles and a reduced ability to produce oocytes capable of fertilization and further development (Fig. 1). The pool of primordial follicles present at birth represents the total number of germ cells available to female mammals throughout their reproductive lives [25,26]. As early as 20 weeks of gestation, the ovary of a human female fetus contains 6 to 7 million oocytes, while only 1–2 million viable oocytes are present at birth [27]. At this stage, the oocyte undergoes the key steps of DNA replication, homologous chromosome pairing and chromosome recombination. The number of viable oocytes further decreases during childhood to between 300,000 and 500,000 at menarche [28]. The process is irreversible because oogonial stem cells disappear after birth. The oocyte/follicular pool diminishes exponentially with age, with an exceptionally sharp decrease after 37–38 years of age [29]. Moreover, there are many biological pathways thought to be responsible for age-related decline in oocyte quality [30], such as age-related mitochondrial defects, the incidence of embryo aneuploidy [31] and chromosomal missegregation [32,33] that are positively correlated with age.

The ovarian surface epithelium (OSE) in aging human beings, monkeys and rabbits, experiences vigorous villous epithelial processes [34], which increases the risk of ovarian diseases such as ovarian cancer, and thereby may compromise the quality of oocytes. In addition, several genes involved in age-related human oocytes quality decline have been identified, including *TOP2B, FOXO3a* [35], *NAMPT* [36] and *SIRT2* [37]. Mitochondrial function is related to maternal age and the degree of ovarian stimulation in cumulus cells [38]. Young and aged nonhuman primates showed that oxidative damage is a key factor in the decline of ovarian function with age [39], while supplementation with the natural polyphenolic compound resveratrol significantly increases pregnancy and implantation rates by improving mitochondrial function [40], and protects against reproductive aging in mice [41]. In line with this, recent studies have shown that the administration of adipose stem cell-conditioned medium (ASC—CM) at advanced age increases the expression of antioxidant and anti-apoptotic genes, thereby promoting in vitro and in vivo embryonic development, because the antioxidant effect of ASC—CM improves the quality of oocytes in older ovaries [42]. Intriguingly, a report shows that a traditional Chinese medicine therapy moxibustion, which burns compressed herbal powder (mugwort) at acupoints, demonstrated potent protective effect on naturally aging ovaries. Long term moxibustion treatment not only reduces the number of ovarian antral follicles and ovarian fibrosis, but also significantly increases estrogen concentration and inhibits apoptosis of ovarian granulosa cells [43]. There is a need for more specific treatments and further research to delay ovarian aging and improve fertility, especially for elderly primipara.

## 3 Advanced maternal age is associated with endocrine disturbances

As age increases, the endocrine system undergoes significant changes, and various physiological functions gradually decline. Age affects the pattern of hormone secretion produced by the hypothalamic–pituitary–hormone organ axis, and the sensitivity of this axis to negative feedback of terminal hormones is also changed. Along with endocrine effects, calcium, bone metabolism and glucose homeostasis are disrupted by aging [44]. Indeed, the endocrine system plays a powerful role in regulating the occurrence and progression of female fertility [45]. Fertility declines beginning in the fourth decade of life, due to the aging of the female reproductive system and accompanying hormonal changes [46]. Changes in the concentrations of sex steroids, including progesterone, androgens, statins and follicle-stimulating hormone (FSH), also appear to be linked to bone loss in older women [47].

### 3.1 Progesterone

Progesterone secreted by the corpus luteum of the ovary maintains pregnancy during ovulation and the start of pregnancy and must bind to receptors on cells within its target tissue, namely, endometrial cells, to be effective [48]. Progesterone is primarily metabolized by 5α-reductase and 3α-hydroxysteroid oxidoreductase in the liver to produce many metabolites. In women, progesterone is present during the luteal phase of the menstrual cycle and during pregnancy; however, levels vary widely, and it is difficult to determine a progesterone deficiency threshold [49]. A trial using African green monkeys to study the effects of AMA on pregnancy adaptability showed that maternal age negatively correlated with circulating progesterone levels during the third trimester of pregnancy [50]. However, when maternal blood, placental tissue and fetal umbilical cord blood were collected from pregnant women of AMA and steroid metabolites were analyzed by liquid chromatography-tandem mass spectrometry (LC‒MS/MS), most hormones, including progesterone, did not change significantly [51]. In young mice, serum progesterone concentrations decreased between the 15th and 18th days of pregnancy, but this change was not apparent in older mice [23].

Progesterone induces decidualization of the endometrial stroma and regulates the expression of various implantation-related autocrine or paracrine factors. Progesterone levels in AMA pregnancies are inconsistent in both humans and nonhuman primates. Nevertheless, the importance of this finding in the context of altered AMA pregnancy outcomes has yet to be elucidated.

### 3.2 Estrogen

Estrogen, which is considered the female hormone, is secreted mainly by ovarian follicular cells in the form of estradiol. Aromatase is a key enzyme involved in estrogen synthesis in the ovary, and estrogen is produced by this enzyme in two different ways: (1) the conversion of testosterone to estrogen is catalyzed by P450 aromatase (P450 AROM), and (2) androstenedione is converted to estrone in follicular membrane cells by P450arom and then to estradiol by 17β-hydroxysteroid dehydrogenase (17-HSD) [52]. Synthesis is controlled by a series of intracellular signaling pathways, transcription factors, hormone regulation and local factors. Estrogen is inactivated in the liver, converted into estriol and estrone, and combined with glucuronic acid to be excreted in the urine. During pregnancy, the placenta also secretes substantial amounts of estriol. Consistent with human data, AMA is associated with estrogen deficiency during late pregnancy in vervet monkeys [53]. Estrogen plays an important role in the female reproductive system. In the early stages of ovarian development, it can promote the formation and activation of primordium follicles by increasing its receptors and cadherin production [54]. During follicular development, it stimulates the proliferation and differentiation of follicles [55]. In the uterus, estradiol (E2) affects uterine proliferation via progesterone through a complex paracrine pathway mediated by the progesterone receptor [56]. Certain neuroendocrine changes are directly related to age; for example, the concentration of gonadotropin after menopause gradually decreases with age [57,58]. In rodents, estrogen antagonizes stress hormone signals, which are critical to their adaptation to pregnancy and fetal development [59]. In addition, estrogen deficiency in women has a variety of effects on human systems, such as cancellous and cortical bone loss leading to osteoporosis [60], impaired vasodilator function, and increased risks and incidences of hypertension and cardiovascular disease [61,62]. Moreover, estrogen is involved in various brain changes, including cognitive decline (especially learning and memory) [63], sleep effects and mood effects. Advanced age also often coincides with emotional conditions such as depression, although there is no consistent correlation between serum hormone levels and emotional symptoms. Data from two multicenter trials indicated that the use of estrogen replacement therapy ERT (progesterone-free) in older women with depression may improve quality of life and overall improvement [64].

### 3.3 Insulin

Generally, glucose tolerance increases with age due to decreased insulin sensitivity [65] and increased lipid levels. Aging is commonly associated with insulin resistance and hyperinsulinemia [66,67]. On the one hand, hyperinsulinemia could induce insulin resistance by downregulating insulin receptors on the cell membrane and disrupting intracellular signaling of receptors in its target cells. On the other hand, increased insulin secretion and/or decreased insulin clearance may lead to hyperinsulinemia during aging. Insulin-like growth factor 1 receptor (IGF1R) and insulin receptor (INSR) belong to the class II receptor tyrosine kinase superfamily and are necessary for embryo implantation. Removal of INSR and IGF1R resulted in decreased fertility, implantation and decidualization failure [68]. IGF1R has been shown to participate in the uterine growth response, mainly by regulating the proliferation of epithelial cells [69,70]. Under E2 stimulation, mouse uterine stromal cells express and secrete insulin-like growth factor-1 (IGF1), and IGF1R is detected in epithelial cells [71]. The epithelial response is induced by stimulating IGF1 and E2, suggesting a paracrine mechanism of action [72]. Insulin signaling affects the proliferation of E2 and progesterone downstream but does not affect uterine receptivity. However, current animal studies are limited to male animals, so further studies of the role of sex determinants in age-related changes in insulin sensitivity are necessary.

### 3.4 Inositols

Inositol is a 6-carbon polyol that is ubiquitous in cells and is mainly endogenously synthesized by the kidney [73]. Some inositol derivatives (e.g., inositolphosphoglycan IPG) are insulin mimetics and are also involved in the regulation of membrane fluidity, protein transport and transport processes, cytoskeleton assembly, intracellular calcium, lipid metabolism and gene expression [74,75]. Inositol has an important physiological role in the placenta. The decrease in placental inositol content in gestational diabetes may be partially mediated by the downregulation of glucose-induced placental inositol synthesis and input, increasing with the elevation of maternal blood glucose. Changes in the level of placental inositol may lead to the pathophysiology of gestational diabetes.

### 3.5 Melatonin

Melatonin (N-acetyl-5-methoxy-tryptamine) is a natural hormone secreted mainly by the pineal gland. It has well-known effects of stimulating antioxidant enzymes [76,77] and anti-inflammatory genes [78], which can eliminate oxidative stress in inflammatory tissues, prevent cell degeneration [79] and regulate circadian rhythm [80]. It is also generally considered an antiaging agent because of its cell-protective effects as an antioxidant.

In females, the effects of melatonin on reproductive physiology are mediated in part by its specific receptors at the hypothalamus, pituitary, and ovarian levels [81,82]. As an antioxidant in follicles [83,84], it reduces oxidative stress and protects oocytes and granulosa cells by removing ROS produced in follicles during ovulation [84]. The abundance of endogenous melatonin decreases with age, leading to excessive accumulation of ROS and DNA damage, triggering apoptosis of aging oocytes [85], which is closely related to the SIRT1 pathway [86]. In mice, melatonin has been found to delay ovarian aging through antioxidant effects, telomere maintenance, stimulation of SIRT expression and ribosomal function, and reduction in autophagy [87]. Clinical data also suggest that melatonin can be used as a dietary supplement to help older women with poor oocyte quality and low ovarian reserve improve fertility outcomes [88,89]. However, these results only raise the possibility of delaying ovarian aging. More research is needed to clarify the molecular mechanisms by which melatonin affects ovarian aging.

Currently, hormone replacement therapy (HRT) is usually used to treat AMA associated hormone disorders clinically, but it may increase the risk of breast cancer, ovarian cancer and cardiovascular disease [90].

## 4 Effects of AMA on placenta

The placenta is a joint organ consisting of mother–child tissue formed by the growth of embryonic membrane and maternal endometrium during human pregnancy. The fetus develops in the uterus, relying on the placenta to obtain nourishment from the mother, while the two sides maintain considerable independence. It serves as the interface between the fetus and the maternal environment and is required for the exchange of gases, nutrients and waste between mother and baby.

Although studies have investigated the impact of AMA on pregnancy outcomes, reports on the histological findings of these placentas are lacking. Whether the increase in perinatal incidence is associated with AMA can be assessed only with an in-depth histopathological evaluation of the AMA placenta and investigation of any potential pathological changes in the AMA placenta [91]. AMA has been suggested to increase the risks of placental abruption, placenta previa [92,93], and uterine bleeding of unknown etiology [94]. Moreover, AMA alters the placental phenotype based on sex-specific factors, thereby impacting the health outcomes of female and male offspring [95] and reducing placental efficiency and placental function [96]. Animal studies suggest that α-Klotho is a potent aging suppressor; overexpression of α-Klotho reduces the aging phenotype and extends the lifespan of mice [97]. Compared with wild-type mice, Klotho-deficient mice are smaller, and its growth hormone show significant atrophy and a decrease in the number of secreted granules [98]. Premature senescence of the placenta leading to adverse perinatal outcomes is caused by AMA [99]. However, to date, few studies have specifically and comprehensively specified the histopathological findings in these placentas.

## 5 AMA increases the incidence of adverse perinatal outcomes

AMA is a strong independent risk factor for adverse pregnancy outcomes [100]. The risk of adverse pregnancy outcomes such as miscarriage, neonatal death, preterm delivery and fetal chromosomal abnormalities usually increases sharply with age in parturients [101]. Such complications could be the cause of preterm birth and increase the risk of perinatal mortality [102]. In addition, older mothers (very advanced maternal age VAMA ≥ 45 years) have been identified as a higher risk group for both mother and fetus [103], with increased risks of preterm delivery and pregnancy-induced hypertension in particular. Furthermore, there is an even higher risk of adverse outcomes during delivery hospitalizations among women aged 45 years and older [104]. AMA was not independently associated with the risk of low birth weight (2500 g) or preterm birth (37 weeks) in Finland [105]. However, if the risks associated with AMA are compared with those associated with smoking, overweight or obesity, there is evidence that the risk of adverse pregnancy outcomes is comparable [106]. Therefore, pregnancies in women over 40 years should be considered as at risk and carefully monitored with individualized care protocols.

### 5.1 Miscarriage

The most common cause of nonrecurrent pregnancy loss is fetal chromosomal abnormalities, some of which are related to maternal age [107]. Recurrent implantation failure (RIF) is defined as the loss of three or more normal embryos before 20 weeks of pregnancy and occurs in approximately 1% to 2% of women [108]. This is often the reason for infertility among AMA women seeking IVF treatment [109]. The integrity of the decidua is a prerequisite for successful implantation and establishment of pregnancy, in addition to endometrial function and embryo quality, which are essential specifically to implantation [110]. There is evidence that decidual defects occur in patients with RIF [111]. However, the molecular mechanisms and potential therapeutic targets of impaired decidualization need to be elucidated. This process begins as an acute stress response, resulting in the differentiation of endometrial stromal cells (ESCs) into specialized decidual cells [112]. Acute senescent decidual cells occur when a large number of ESC cells fail to differentiate [113]. The senescence-associated secretory phenotype (SASP) is a state of permanent cessation of the cell cycle and secretion of inflammatory mediators, chemokines, growth factors, and extracellular matrix (ECM) proteases, defined as cellular senescence [114,115]. Acute senescent cells usually eliminate themselves by recruiting innate immune cells (mainly natural killer cells) [116] to reduce tissue inflammation and promote the transformation of the interstitium into pregnancy decidua. The midluteal endometrium in RIF is related not only to the lack of eMSCs but also to excessive decidual senescence based on marker genes of the decidual pathway identified by high-throughput single-cell RNA sequencing [117]. Oral administration of the DPP4 inhibitor sitagliptin (100 mg daily) increased eMSC abundance in RIF patients to improve the *peri*-implantation endometrium, which is associated with a significant reduction in senescent decidual cells [118]. On the other hand, embryo implantation occurs in the context of the maternal immune response and may threaten reproductive success [119]. Therefore, it is generally believed that the immune response of pregnant women should be suppressed to maintain tolerance to allogeneic fetuses. However, this is not the case. The activation of the uterine immune response, including the development of immune tolerance and promotion of angiogenic cells, differs from the traditional immune response in killing invading cells derived from external sources [120]. Second, full activation of the uterine immune response of dNKs and macrophages is necessary for the healthy growth of the fetus. The molecular mechanism and potential therapeutic targets of decidual injury in patients with RIF and the adaptive immune response at the maternal–fetal interface of older women need to be further clarified.

*Recurrent miscarriage (RM):* RM affects millions of couples worldwide, and in approximately half of cases, no clear cause is identified [121]. More than 80% of women aged 45 years or over who become pregnant experience miscarriages [122,123]. Detecting parental chromosomal abnormalities and determining the karyotypes of fetuses from pregnancy losses among women aged ≥36 years [124] are invaluable for minimizing the impact of increased maternal age on the aneuploidy rate among RM-affected couples, as this can exclude aneuploid embryos through IVF and preimplantation genetic testing (for aneuploidy [PGT-A]) [125]. However, the beneficial effects of aneuploidy screening seem to be more pronounced among patients younger than 37 years of age [126].

### 5.2 Preterm birth

Preterm birth (PTB) is usually defined as regular contractions at less than 37 weeks of pregnancy with cervical changes and accounts for up to 75% of perinatal mortality. The pathogenesis of PTB is not clear, but PTB may represent the result of early idiopathic activation or pathological damage during normal delivery. At present, PTB is considered a syndrome caused by multiple mechanisms, including infection or inflammation, uterine–placental ischemia or hemorrhage, uterine overdilation, stress and other immune-mediated processes. The link between preterm delivery and AMA remains controversial. Previous studies have reported an association between AMA and preterm labor [127]. Various studies have attempted to study the relationship between older women and premature delivery (spontaneous and iatrogenic) under a variety of confounding factors, but the evidence is still conflicting [128]. Some authors report that after adjusting for confounding factors, pregnancy in older women (≥40 years) is associated with an increased risk of PTB. Specifically, PTB occurs mainly among young pregnant women (20–24 years old) and women over 40 years old [129]. Based on a cohort study of a group of Danish women, there was a U-shaped association of maternal age with the risk of PTB. The minimum PTB risk occurs from the age of 24–30 years [130]. More specifically, a registration-based national cohort study conducted in Finland found that the threshold age for PTB is 28 years of age [131]. Further research is warranted to determine whether AMA is an independent factor in PTB [132].

### 5.3 Gestational diabetes mellitus

Gestational diabetes mellitus (GDM) is traditionally defined as carbohydrate intolerance of varying severity that develops or is first detected during pregnancy [133]. The most recent definition of GDM is diabetes diagnosed in the second or third trimester that was not evident prior to pregnancy [134]. It is the most common metabolic disorder during pregnancy and can endanger maternal and infant health.

GDM is characterized by insulin resistance (IR) accompanied by failure of islet β cell compensation [135]. Noticeably, AMA pregnant women have higher IR levels of anti-HOMA [136]. It has been reported that the incidence of GDM increases with maternal age, reaching a plateau around the age of 40 [137]. Notably, the incidence of GDM is still significantly higher in AMA pregnancies after adjusting for pregnancy, educational level, blood relationship, and cesarean section rate [138]. Accumulative evidence has revealed that the etiology of GDM is complex, with mechanistic and epidemiological studies showing the involvement of genetic and environmental factors, and there are significant interactions between maternal age, body mass index (BMI), ethnic origin and GDM incidence [139]. For instance, hyperglycemia is associated with acute thallium (TI) poisoning. Intestinal trace elements such as TI may lead to glucose metabolism disorder and increase the risk of GDM during pregnancy. This association is partly mediated by prepregnancy BMI, with older age and higher prepregnancy BMI associated with higher serum thallium concentration (STC) levels [101,140], especially in Black African and South Asian women.

### 5.4 Fetal growth restriction (FGR)

Although its continuous function is a necessary condition for successful pregnancy, the placenta ages as the pregnancy progresses. A strong correlation between placental trophoblast aging and FGR pregnancy has been reported [141]. Placentas affected by FGR have poor vascularization, as well as decreased vascular branches and capillaries [142]. Increased apoptosis [143], decreased proliferation [142] and increased syncytial nuclear aggregates (SNAs) are also evident in the placentas of pregnancies with FGR [144]; however, it is not clear whether there are changes in the development, structure or function of the placenta of AMA mothers and whether they are the basis of FGR susceptibility. In an aged (40-week-old) mouse model, there was histological evidence of obstruction of uterine artery remodeling and changes in maternal uterine vascular function [145]. However, this study did not analyze the function of the placenta. More extensive research is needed to explore the relationship between AMA and placental and uterine–placental dysfunction.

### 5.5 Preeclampsia

PE is a common condition of pregnancy, marked by the onset of hypertension and proteinuria [146]. Preeclampsia is associated with abnormal trophoblast (blastocyst cells) invasion of the uterus and uterine spiral arteries [147], reduced placental perfusion [148], an imbalance between proangiogenic and antiangiogenic factors [149] and an excessive intravascular inflammatory response to placental tissue. The molecular and cellular mechanisms that cause PE are still unclear. This is partly due to the lack of a preclinical model that is physiologically related to human pregnancy and allows for the control of environmental and genetic variability inherent in human research. Advanced age significantly increases the incidence of PE: studies have shown that women over 40 years of age have a 1.5–2.0 times higher risk of PE than younger women, whether they are primiparous or multiparous [150]. A model with maternal age as the competing risk continuous variable could help clinically minimize unexpected perinatal adverse events. The model was screened during the first trimester of pregnancy to identify a high-risk group that would benefit from preventive treatment interventions. Screening during the second and third trimesters to identify high-risk groups will facilitate close monitoring of the early diagnosis of preeclampsia [151].

In addition, increased aging of the placenta or trophoblasts in preeclampsia has been confirmed. In the third trimester of pregnancy, villi that have reached the end of their lives have enlarged stromal chambers, and fewer CTB and STB show signs of aging. Placental oxidation and endoplasmic reticulum stress [152] can lead to cell aging, which may lead to this pregnancy complication. In severe preeclampsia, there is significant reprogramming of the cytotrophoblast epigenome during pregnancy, especially the overall significant increase in H3K27 acetylation of trophoblast cells, which means that epigenetic changes are not downstream of DNA sequence changes, unlike many cancer types [153].

### 5.6 Intrahepatic cholestasis of pregnancy

Intrahepatic cholestasis of pregnancy (ICP) is the most common pregnancy-specific liver disease and is associated with an increased risk of adverse perinatal outcomes, including spontaneous preterm delivery, meconium staining of the amniotic fluid, and stillbirth. It is characterized by itchy skin and elevated serum bile acid levels. Severe cases lead to intrauterine fetal death. Ursodeoxycholic acid (UDCA), which can relieve pruritus and reduce bile acid levels, is the most common obstetrical prescription drug for ICP, but its beneficial effect on fetal outcomes is controversial [154]. The cholagogic effect of UDCA may be mediated by its activity on MRP2 or MRP3 receptors. Other drugs that may enhance the efficacy of UDCA, such as rifampicin, can inhibit bile acid reuptake by hepatocytes and enhance the detoxification of bile acids. More effective drugs still need to be developed. In addition to the most important risk factors, namely, hepatitis C virus (HCV) [155,156], seasonal onset (winter) [157], low selenium levels [158], low vitamin D [159], and multiple pregnancies [160], advanced age has also been proven to increase the risk of ICP [161].

### 5.7 Peripartum cardiomyopathy (PPCM)

PPCM is a potentially life-threatening pregnancy-related disease that usually occurs during the perinatal period and is marked by left ventricular dysfunction and heart failure. To meet the needs of the rapidly developing fetus, the mother will undergo physiological cardiovascular adaptation, such as a 50% increase in blood volume during pregnancy, an increase in cardiac output, and a 75% increase in cardiac output during delivery [162]. These large hemodynamic changes increase cardiac load throughout pregnancy, which can lead to myocardial infarction/ischemia, acute pump failure, or cardiac arrest in older women [127,163]. Studies have shown that maternal age is an important predictor of cardiovascular function, such as cardiac output, total peripheral resistance, stroke volume, and ventricular ejection time [164]. Moreover, the age of pregnant women is an independent factor affecting the decline in diastolic function when the left ventricular ejection fraction is normal. Either delete this part of the sentence or add clarity: ‘with an inflection point in the relationship between diastolic function and maternal age of 32.5 years’ [165]. Previous case reports have identified clinical overlap between familial dilated cardiomyopathy (DCM) and PPCM, although the extent of overlap is largely unknown. Approximately 15% to 20% of PPCM patients carry mutations that cause cardiomyopathy, mainly in the TTN, MYH7 and SCN5A genes. Mutations in these genes are also associated with dilated cardiomyopathy [166]. Myocardial infarction is becoming more common in late pregnancy and the perinatal period, in part due to maternal age and other lifestyle changes [167]. Ischemia/reperfusion injury models have been successfully established in rodents, showing that the area of myocardial infarction caused by ischemia‒reperfusion injury in late-pregnant rodents was approximately fourfold that of nonpregnant controls [168]. In addition to our current limited understanding of age-related heart disease in pregnancy, cardiometabolic changes in pregnant women are also crucial, and understanding these changes may have significant clinical implications.

Since there are no effective treatments specifically lowering the perinatal risks for AMA pregnancies, accurate dating, and counseling on aneuploidy screening, with consideration for early diabetes screening, should be performed in the first trimester. A detailed anatomy scan and fetal echocardiogram should be completed by 22 weeks' gestation, along with routine and high-risk (if indicated) prenatal care. Close attention should be given to the development of pregnancy-related complications associated with advancing age. Third-trimester fetal surveillance can be considered. Given that no contraindications exist, these patients should be encouraged to pursue a vaginal delivery with consideration for induction at 39 to 40 weeks' gestation [169].

## 6 Effects on maternal long-term health

The rising rates of AMA will have an overall impact on women across the age spectrum. Although data specifically pertaining to women of advanced age are limited, there is some evidence to support the concept that maternal long-term health is affected by age at parturition.

### 6.1 Postpartum depression (PPD)

*PPD* is becoming a global health concern. During pregnancy and the postpartum period, the stress response can lead to hypercortisolemia [170]. Numerous studies have shown that cortisol overproduction increases susceptibility to depression [171]. One study showed that mothers become less prone to developing PPD as maternal age increases [172], and this probability changes according to the number of children at home. In contrast to this finding, another recent study reported that postpartum depression affects women of AMA at higher rates [173]. Selective serotonin reuptake inhibitors (SSRIs) such as paroxetine, fluoxetine, and sertraline are most commonly medication for PPD. Recently, clinical trials have shown that intravenous injection of Brexanolone (Zulresso) can alleviate symptoms of postpartum depression within hours [174]. The FDA approved Brexanolone injection for the treatment of PPD in 2019 makes it the first medication in history to specifically target PPD.

### 6.2 Pregnancy and lactation-associated osteoporosis (PLO)

This is a rare disease with little known about its pathophysiology, yet it typically occurs in late pregnancy and early lactation [175]. AMA is one of the higher risk factors [176]. PLO affects mainly the vertebral body and hip joints [177]. Many patients may have vertebral fractures and even kyphosis [178]. PLO places heavy physical and psychological burdens onto patients, affecting their quality of life and work ability. Although the etiology and pathophysiology of PLO have not been widely studied and its diagnosis and treatment are still controversial, it can be treated by teriparatide and zoledronic acid [179], as well as supplement of alendronate, calcium carbonate and vitamin D.

### 6.3 Pelvic floor dysfunction (PFD)

With the increasing proportion of pregnancies associated with AMA, pelvic floor dysfunction (PFD) also warrants greater attention. PFD is caused by factors such as degeneration of, and injury to, the supporting tissues of the pelvic floor, and can lead to urinary incontinence, chronic pain, pelvic organ prolapse, and sexual dysfunction, among other problems. However, the natural history and mechanisms of PFD in older women have not been well understood or explored. Some cumulative effects, such as menopausal estrogen deficiency, higher BMI, previous pelvic surgery and a history of diabetes and hypertension, can affect the function and structure of the pelvic floor in older women [180], thus a longitudinal study is needed to explore this problem. PFD can also cause psychological distress related to inferiority complexes, depression, and other conditions, which further threaten physical and mental health [181]. In view of the relatively poor anatomical and functional results of perineal injury and anal sphincter tear repair during vaginal delivery for women of AMA, it has been found that the risk of pelvic floor injury increases with maternal age [182]. However, this connection is not significant [183]. Sphincter injuries may be more common in older women who have their first child, at least in some populations [182,184], which may be an important factor when evaluating risks associated with vaginal delivery as opposed to cesarean section.

## 7 Discussion and conclusion

In both developed or developing countries, aging populations pose challenges in all aspects. To effectively respond to such challenges, some countries have formulated intervention strategies that suit their own national conditions (for example, the two- and three-child policy implemented in China). To develop antiaging intervention strategies, it is necessary to understand the aging characteristics and molecular mechanisms of different tissues and organs [185]. As countries discover an equilibrium between replacement level fertility policies, socioeconomic development, and optimizations in healthcare, the next few decades may yield substantial changes in productivity, economic development, social benefits, and living standards. These changes will substantially impact reproductive possibilities.

However, these visions of population growth and balance continue to depend on the fertility of women. The decision to delay childbirth is becoming increasingly common among global populations, as individuals pursue economic independence, career aspirations, stable housing and improved lifestyle attributes. These rising rates of delayed childbirth have uncovered a variety of previously unrecognized risks of AMA-associated pregnancy and parturition. In this review, we updated the current understanding of maternal and fetal outcomes associated with AMA (Fig. 1). However, the pathogenic mechanisms underlying the increased associated risks remain largely unknown. There is a need to identify underlying causes of associated risks as well as technologies and interventions that can circumvent risks or otherwise support AMA-associated pregnancies. Support strategies must include prepregnancy screening, interventions, and postpartum maintenance, with the broad goal of reducing the impact of aging, extending the reproductive age span of women, and delaying the onset of menopause. Necessarily, this research will involve seeking effective antiaging interventions between puberty and menopause.

The population undergoing ART and frozen embryo transfer (FET) is increasing; despite that these technological developments that contribute to a widening window of female reproductive capacity, maternal age remains an important factor with varying effects on maternal and fetal/infant health. In recent years, new intervention strategies have been identified for aging cell therapies, such as regulators of the anti-inflammatory response, oxidative stress, DNA damage and mitochondrial and protein dysfunction, to prevent or even reverse female reproductive aging and improve the health of offspring [186]. At the same time, scientific research continues to interrogate functional mechanisms of healthy maternal and child outcomes as well as to support technologies. Further advances will be vital to developing promising strategies to treat low fertility or infertility in older women and extend the span of reproductive potential in humans.

## Declaration of competing interest

The authors declare that they have no conflicts of interest in this work.
